# Case of Phthisis, with Pneumothorax and Empyema, in an Infant, Aged Seven Months

**Published:** 1854-03

**Authors:** W. F. Cleveland


*Case of Phthisis, with Pneumothorax and Empyema, in an Infant, aged Seven Months.* By W. F. Cleveland, Esq.—On the 15th of February, 1853, W. J. M., a delicate and strumous-looking child, aged 5 months, was brought to my house, suffering from cough and slight dyspnœa,—symptoms which, his mother stated, he had had, in a greater or less degree, ever since he was a week old.

His father had been under my care, and died, about a month previously, of advanced phthisis.

A mixture, containing ipecacuanha, squills, and syrup of poppies, was prescribed; and, as he soon became better, I saw no more of him, until the 9th of March, when he was greatly distressed, and appeared to be suffering from some abdominal uneasiness, as there were continued efforts of straining or tenesmus.

A carminative mixture, to be followed by a dose of castor-oil, was given, and on the following day he was relieved.

In the course of a few days, however, the cough became more troublesome, and was attended with marked dyspnœa. He now seemed to be in great pain, and was constantly crying, so that, although more than one attempt was made to learn the state of the chest by a physical examination, no satisfactory conclusion could be arrived at.

He continued to grow weaker, and suffer greatly from the cough, want of sleep, night-sweats, etc., until the 7th of April, when it was thought he was fast sinking; but, contrary to expectation, he rallied, and for some days was better than usual.

On the 6th of May, there was great aggravation of all the symptoms; he had passed a very restless night, and once or twice his mother thought he would have been suffcated. During my visit in the course of the day, a paroxysm of coughing came on; he made a few convulsive efforts to expectorate, and death almost immediately followed.

With the assistance of my friend, Mr. Burford, I examined the body, twenty-one hours after death.

*I have since heard that this is true, and that five dead bodies were thrown overboard in the roadstead, and it was at first believed that the illness arose from eating fish which had preyed on the bodies. The people will not touch fish.

There was no remarkable emaciation. On removing the sternum, the right pleural cavity presented the fallacious appearance of a large vomica, being about one-third full, and containing about four ounces of slightly fetid, thick, greenish pus. The walls of this cavity were thick, and lined here and there with flakes of effused fibrin.

At about the junction of the middle with the lower third of the sac, towards the vertebral column, was an elliptical opening, about the size of a small horse-bean, through which a probe could be readily passed, in an upward and backward direction, into a large bronchial tube. Under the margin, which was smooth and rounded, of this pleural opening was an excavation in the lung, as large as a common filbert. The lung itself was condensed, and lay on the spine.

On a more careful examination, crude tubercles were found scattered throughout it, and, in the cavity above mentioned, as also in the bronchial tube, there was purulent matter similar to that found in the pleural sac.

The left lung contained tubercles, but none of them could be said to have decidedly softened. The heart and other organs were healthy.

One of the most interesting questions connected with the foregoing case, but one, it must be admitted, that cannot now be satisfactorily determined, is, whether the aperture was caused by the pointing of an empyema into the lung, or the extension of a vomica into the pleura. Still, setting aside the fact, that, in the large proportion of recorded cases of pneumothorax the latter state obtains, there is strong presumptive evidence that this child was also first affected with phthisis, and that the empyema was secondary, or consequent on the bursting of the vomica into the pleural cavity. Nor does experience prove that the circumstance of the non-softening of tubercles in other parts of the lungs should be allowed to invalidate this conclusion.

We observe, then, a remarkable hereditary predisposition to phthisis, as shown in the death of the father, in an advanced stage of that disease, when the child is only four months old. There is also the history of a cough, with shortness of breath (indicating the existence of tubercles,) commencing a week after birth.

Then, after these symptoms have continued nearly six months, suddenly arise what were at the time, supposed to be obscure indications of abdominal uneasiness, but which are now explicable, as referring to the ordinary constitutional distress, including the hurried respiration, etc., produced by pneumothorax. A few days after this event, aggravation of cough, increased dyspnœa, with signs of pain, announce the development of pleuritis with effusion.

The subsequent progress, with the exception of but a short interval, is that of increasing debility, hectic, cough with expectoration, etc., until death somewhat suddenly takes place from suffocation following the ineffectual efforts to expectorate, in all probability, some of the viscid, purulent matter that had escaped from the pleural cavity.

I will only remark further, that had the empyema opened into the lung, it could scarcely have failed to be known at the time by the expectoration of some of its contents, and that the existence of a tubercular cavity immediately under the surface of the pleura, as was here found after death, when taken in conjunction with the progress of the case during life, is an argument in favor of the theory I have endeavored to substantiate.—*London Medical Times and Gazette.*

